# Understanding the factors affecting the humanitarian health and nutrition response for women and children in Somalia since 2000: a case study

**DOI:** 10.1186/s13031-019-0241-x

**Published:** 2020-05-27

**Authors:** Zahra Ahmed, Anushka Ataullahjan, Michelle F. Gaffey, Mohamed Osman, Chantal Umutoni, Zulfiqar A. Bhutta, Abdirisak A. Dalmar

**Affiliations:** 1 Somali Disaster Resilience Institute, Mogadishu, Somalia; 2 Centre for Global Child Health, The Hospital for Sick Children, Toronto, Canada; 3 UNICEF Somalia, Nairobi, Kenya; 4 Centre of Excellence in Women and Child Health, Aga Khan University, Karachi City, Pakistan

**Keywords:** Somalia, RMNCH, Reproductive health, Maternal health, Newborn health, Child health, Adolescent health, Nutrition, Conflict, Humanitarian

## Abstract

**Background:**

Somalia has been ravaged by more than two decades of armed conflict causing immense damage to the country’s infrastructure and mass displacement and suffering among its people. An influx of humanitarian actors has sought to provide basic services, including health services for women and children, throughout the conflict. This study aimed to better understand the humanitarian health response for women and children in Somalia since 2000.

**Methods:**

The study utilized a mixed-methods design. We collated intervention coverage data from publically available large-scale household surveys and we conducted 32 interviews with representatives from government, UN agencies, NGOs, and health facility staff. Qualitative data were analyzed using latent content analysis.

**Results:**

The available quantitative data on intervention coverage in Somalia are extremely limited, making it difficult to discern patterns or trends over time or by region. Underlying sociocultural and other contextual factors most strongly affecting the humanitarian health response for women and children included clan dynamics and female disempowerment. The most salient operational influences included the assessment of population needs, donors’ priorities, and insufficient and inflexible funding. Key barriers to service delivery included chronic commodity and human resource shortages, poor infrastructure, and limited access to highly vulnerable populations, all against the backdrop of ongoing insecurity. Various strategies to mitigate these barriers were discussed. In-country coordination of humanitarian health actors and their activities has improved over time, with federal and state-level ministries of health playing increasingly active roles.

**Conclusions:**

Emerging recommendations include further exploration of government partnerships with private-sector service providers to make services available throughout Somalia free of charge, with further research on innovative uses of technology to help reaches remote and inaccessible areas. To mitigate chronic skilled health worker shortages, more operational research is needed on the expanded use of community health workers. Persistent gaps in service provision across the continuum must be addressed, including for adolescents, for example. The is also a clear need for longer term development focus to enable the provision of health and nutrition services for women and children beyond those included in recurrent emergency response.

## Background

Routinely characterized as a ‘fragile state’, Somalia has been ravaged by more than two decades of active fighting that has caused immense damage to the country’s infrastructure and mass displacement and suffering among its people. An influx of humanitarian actors has sought to provide basic services, including health services for women and children, throughout the conflict. At present, there are some 328 humanitarian organisations operating in Somalia [1], contending with ongoing insecurity and recurrent natural disasters.

**Fig. 1:**
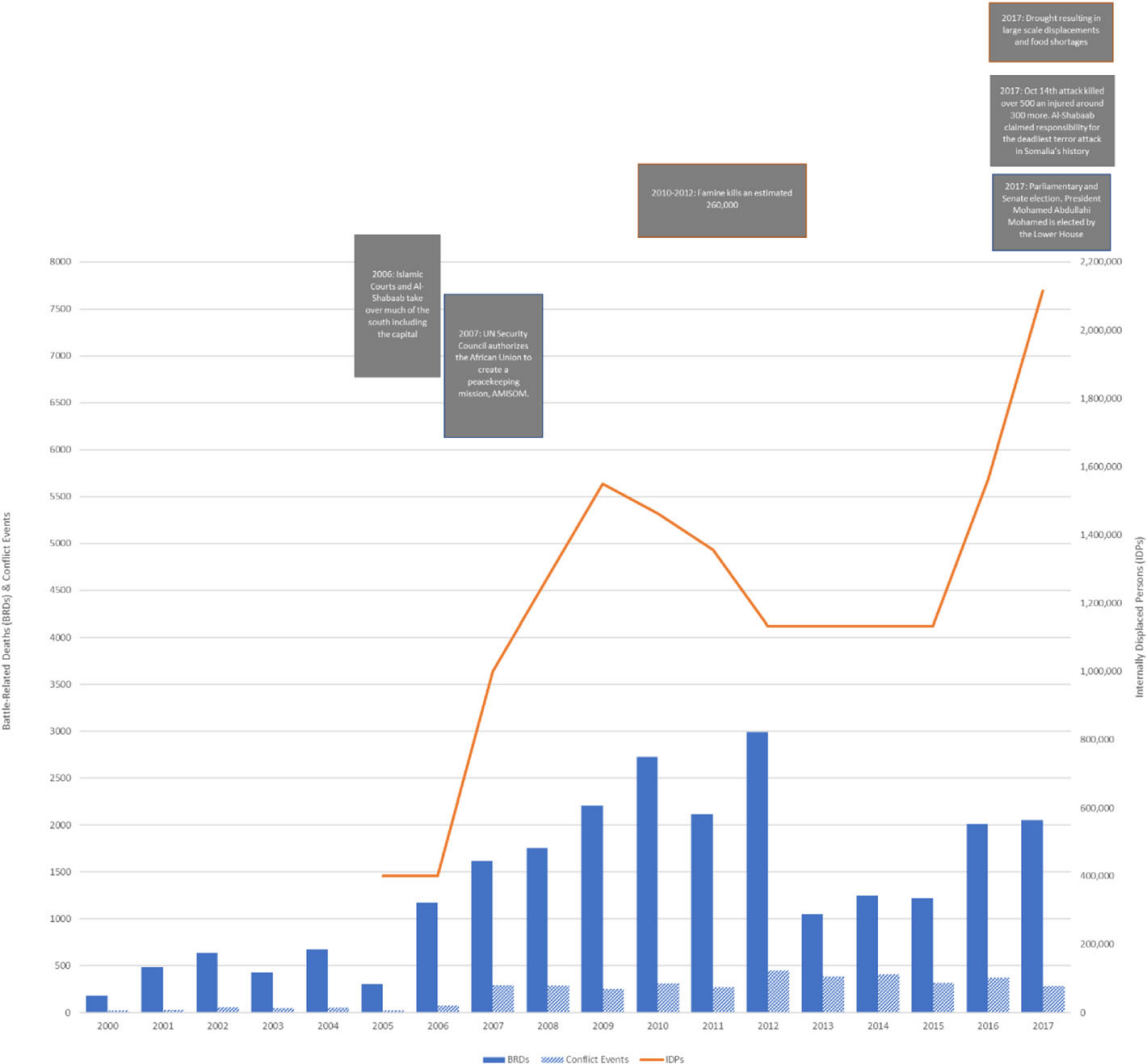
Battle-related deaths, conflict events, and internally displaced persons in Somalia, 2000–2017 Death and event estimates are sourced from the Uppsala Conflict Data Program [2, 3]; IDP estimates are sourced from the Somalia 2019 Humanitarian Response Plan [1]

The contemporary conflict in Somalia has a long history. After decades of military dictatorship under Mohamed Siad Barre, a long-running civil war erupted following the military coup that ousted him in 1991. Mortality directly attributable to armed conflict in Somalia peaked in 1991, with some 8000 battle-related deaths (BRDs) reported, and at least 1000 BRDs have been reported every year since the formation of the Al-Qaeda-affiliated Al-Shabaab (Arabic for ‘The Youth’) in 2006 [2]. Elevated mortality has also been coupled with massive, prolonged displacement (Fig. 1). Though the militant group’s forces have been pushed out of the main cities in the last decade due to a concerted effort by Somali national armed forces and the African Union Mission in Somalia (AMISOM), they maintain strongholds in Central and South Somalia and high profile attacks continue in cities such as Mogadishu and Baidoa. Recurring droughts have further exacerbated the humanitarian situation. At present, over 2.6 million people are internally displaced due to conflict and drought [1].

Health and nutrition indicators in Somalia are some of the worst in the world. Recent estimates suggest that the rates of global acute malnutrition are approximately 14%, with even higher rates among internally displaced populations (IDPs) and pastoralist communities [4]. According to Save the Children’s 2015 *State of the World’s Mothers* ranking of 179 nations, Somalia is the worst place in the world to be a mother, while UNICEF’s 2017 *State of the World’s Children* report identified Somalia as having the highest under-5 mortality rate in the world [5, 6].

Under-5 mortality stands at 133 deaths per 1000 live births, infant mortality stands at 83/1000 and neonatal mortality stands at 39/1000 [6].

Delivering health services to populations in need in Somalia is particularly complex given the territory’s governance structure. Although Somalia currently includes Federal Member States Jubaland, South West State, Hirshabelle, and Galmudug in addition to Puntland and Somaliland, Somaliland declared independence in 1991 and Puntland declared autonomy in 1998. Somaliland has had functioning governing structures since the collapse of the central government in 1991, but the region is not recognised an as independent by the Federal Government of Somalia. The role of the Federal Government in Somaliland and Puntland is controversial, and navigating these complex dynamics can be difficult for humanitarian agencies.

Moreover, the absence of government throughout the civil war and the subsequent years of widespread conflict resulted in the deterioration of Somalia’s public sector health system to virtual non-existence. This gap left space for the private sector to proliferate, largely unregulated, to provide health and nutrition services to the population. Existing health infrastructure such as regional hospitals were taken over by international NGO service providers and have only recently returned under government control. Public sector health facilities were mapped and assessed by WHO in 2016 [7], and although the private health sector was assessed by the UK’s Department for International Development in 2015, it remains unregulated despite the increasing capacity of the federal government with no formal public-private partnerships [8]. Currently, private sector providers act primarily as referral points for beneficiaries from government and NGO-run facilities.

At present, the Federal Government of Somalia is working to increase its oversight of health services in the country. In 2014, health service provision was consolidated into a four-tier approach under the Essential Package of Health Services (EPHS). This framework normatively outlined the standard treatment guidelines for a comprehensive range of health services available to the public for free [9]. More recently, the federal government has also developed the first national plan in 30 years, the *National Development Plan 2017–2019*, and is in the process of drafting the plan for 2020–2024 [1].

Against this background, the study reported here aimed to better understand the humanitarian health response for women and children in Somalia since 2000, including what services were planned and prioritized in this complex environment, how, and why.

## Methods

The study presented here is part of a multi-country study coordinated by the BRANCH Consortium [10] and focused on the delivery of reproductive, maternal, newborn, child and adolescent health (RMNCAH) and nutrition interventions in ten conflict-affected countries: Afghanistan, Colombia, Democratic Republic of the Congo, Mali, Nigeria, Pakistan, Somalia, South Sudan, Syria, Yemen [10]. This study employed a mixed-methods design, including both secondary analyses of existing quantitative data and primary qualitative data collection and analysis. The quantitative component aimed to examine trends in the coverage of RMNCAH & nutrition interventions in Somalia since 2000, as well as conflict-related displacement and conflict-attributable mortality. The qualitative component aimed to better understand the coordination and decision-making processes of humanitarian actors delivering health services during this period, particularly in Bay region and in the capital Mogadishu in Benadir region, in the Central-South Zone of Somalia.

### Data collection

#### Quantitative

Estimates of intervention coverage of RMNCAH and nutrition interventions in Somalia over time were extracted from reports of large-scale household surveys conducted since 2000 and available in the public domain: the 2006 and 2011 Multiple Indicators Cluster Surveys (MICS), the 2009 National Micronutrient and Anthropometric Nutrition Survey (NMANS), the 2016 Service Availability and Readiness Assessment Survey (SARA), and the 2017–2018 Somali High Frequency Survey (SHFS). Where relevant estimates were not published but the dataset was available, we generated estimates from the data, accounting for survey design. Estimates of deaths directly attributable to conflict in Somalia over time were derived from publicly available data from the Uppsala Conflict Data Program [2, 3]. Regional estimates of the cumulative number of internally displaced people were extracted from the Somalia 2019 Humanitarian Response Plan [1].

#### Qualitative

Primary qualitative data collection was conducted between August and October 2018 in Mogadishu and Baidoa in Somalia and in Nairobi, Kenya. Interview guides were informed by shared templates created by the BRANCH consortium. Through a collaborative process, the research team adapted these guides to reflect the local context [10].

A team of five local data collectors (four men and one woman) was utilised to conduct in-depth interviews. All the data collectors held advanced degrees in public health or the social sciences and had previous experience collecting qualitative data. They also had an in-depth understanding of public health issues in Somalia. They were bilingual in Somali and English which allowed them to conduct interviews in either language, depending on the respondents’ preferences.

Interviews were conducted with local NGO staff, international NGO staff, MOH staff, and private health facility staff. Respondents included a range of individuals such as country directors, program managers, health officers, and project coordinators, and were purposively sampled through three strategies. Target organizations for key informant interviews were identified by the study team’s review of “Who does What Where (3W)” matrices produced by the UN Office for the Coordination of Humanitarian Affairs (OCHA), and specific respondents were identified through discussion with organizational representatives about which individuals would best be able to speak to our research objectives. Nominations were also solicited from organizational and government representatives attending a project inception meeting convened by the study team in Nairobi in 2017. Snowball sampling was also used, whereby participating respondents suggested other potential key informants to the study team. Initial contact was made via phone or email depending on the contact information available for the respondent.

A total of 27 interviews were conducted face-to-face in the respondent’s offices while 6 interviews were conducted via Skype as the respondents were in geographies the study team could not access. For interviews conducted via Skype, informed consent was provided by participants verbally at the start of the interview, while those participating in in-person interviews provided informed consent through a signed consent form. No invited participants refused to give consent and all agreed to have the interviews recorded. Interviews lasted approximately 60–90 min (Table 1).

**Table 1:** Respondent information

Administrative Focus	UN Agencies	International NGOs	Local NGOs	Ministry of Health	Private Health Providers	Health Facility Staff	Total
National	2	10	1	3	1	2	19
Regional	1	3	6	1	–	2	13

### Data analysis

#### Quantitative

We tabulated quantitative estimates of achieved coverage of key health and nutrition interventions for women and children, stratified by zone (the lowest level of aggregation for which representative estimates could be derived from national survey sampling approaches): Northwest Zone/Somaliland; Northeast Zone/Puntland; and Central-South Zone. Time trends in the frequency of battle-related deaths and lethal violence events were generated nationally.

#### Qualitative

Key informant interview transcripts were imported into NVivo 12 software. Latent content analysis, an inductive analysis approach, was used to analyze the data. First, ZA and AA read the interview transcripts, and then coded passages according to the speaker’s meaning. ZA then merged the codes into categories while ensuring that they had both internal homogeneity and external heterogeneity [11]. These categories were later merged into themes shared in this manuscript. Coding and analysis was undertaken in an iterative manner, so that new ideas that emerged could be probed in additional interviews. Peer debriefing with the analysis team also assisted in identifying areas to further investigate during interviews. Member checking was conducted through several consultations with stakeholders for their input and feedback.

## Results

### Achieved coverage of RMNCAH and nutrition interventions

**Fig. 2:**
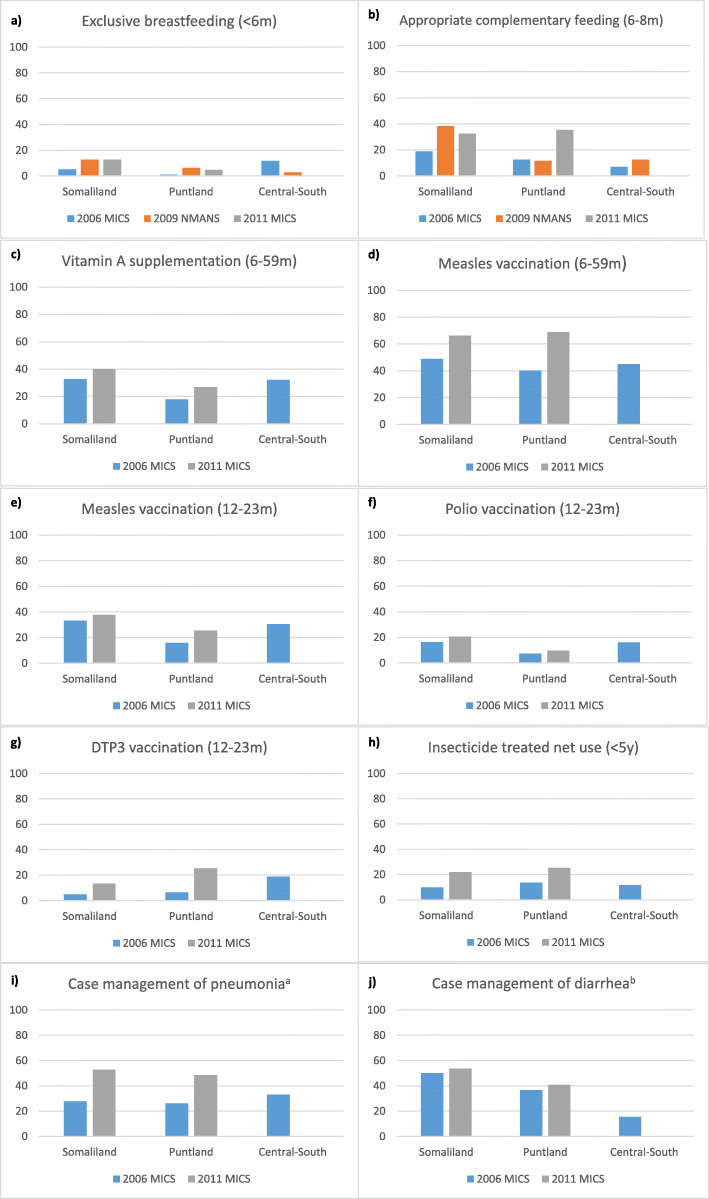
Representative household survey estimates of child health and nutrition intervention coverage, by zone. **a** Percent of children <5y with suspected pneumonia treated with antibiotics. **b** Percent of children <5y with diarrhea treated with oral rehydration solution

The available quantitative data on RMNCAH and nutrition intervention coverage in Somalia are extremely limited, making it difficult to discern patterns or trends in coverage over time or in different regions. Table 2 presents available coverage estimates for a set of reproductive, maternal and newborn interventions, showing low or very low coverage of most interventions overall, but higher coverage in Somaliland than in Puntland or Central-South for nearly all indicators in 2006, and higher coverage in Somaliland than Puntland for nearly all indicators in 2011. (No 2011 data are available for Central-South.) Coverage of most interventions increased between 2006 and 2011 in Somaliland, with the exception of both modern and traditional contraceptive prevalence rates (CPR), while the traditional CPR as well as both antenatal care indicators (at least 1 visit, at least 4 visits) declined in Puntland between 2006 and 2011.For some interventions implemented during infancy and childhood (Vitamin A supplementation, and measles and polio vaccination), coverage levels in Central-South in 2006 were closer to those achieved in Somaliland than in Puntland. Coverage levels in Central-South were the highest of all three zones for exclusive breastfeeding among children < 6 months, DTP3 vaccination and case management of pneumonia, but the lowest for complementary feeding and case management of diarrhea (Fig. 2). By 2011 (when no data are available for Central-South), coverage levels of all child health and nutrition interventions had increased in both Somaliland and Puntland but were still low or very low overall, except for relatively high measles vaccination coverage among children 6–59 months of age.

**Table 2:** Representative household survey estimates of reproductive, maternal and newborn health intervention coverage, by zone

	Somaliland	Puntland	Central-South
Contraceptive prevalence rate – modern			
2006 MICS	4.6	0.1	0.3
2011 MICS	1.5	0.2	–
Contraceptive prevalence rate – traditional			
2006 MICS	21.0	11.8	11.3
2011 MICS	8.3	2.4	–
Antenatal care – at least one visit			
2006 MICS	39.6	30.2	29.4
2011 MICS	51.2	27.9	–
Antenatal care – at least four visits			
2006 MICS	10.3	5.8	5.2
2011 MICS	14.8	3.3	–
Skilled birth attendance			
2006 MICS	41.3	36.8	29.7
2011 MICS 1	44.1	38.4	–
Institutional delivery			
2006 MICS	21.5	7.9	5.8
2011 MICS	30.6	12.7	–
Caesarean-section rate			
2006 MICS	2.6	1.9	2.0
2011 MICS	12.5	13.4	–
Early initiation of breastfeeding (< 1 h)			
2006 MICS	35.1	38.5	21.4
2011 MICS	60.9	56.0	–

**Fig. 3:**
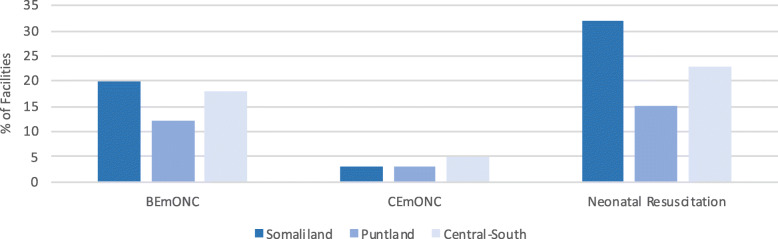
Facility readiness to initiate neonatal resuscitation and provide emergency obstetric and newborn care, by zone, 2016

In 2016, the SARA survey in Somalia showed that facility readiness to provide basic emergency obstetric and newborn care and to initiate neonatal resuscitation was highest in Somaliland, followed by Central-South, while still very low overall (Fig. 3).

**Fig. 4:**
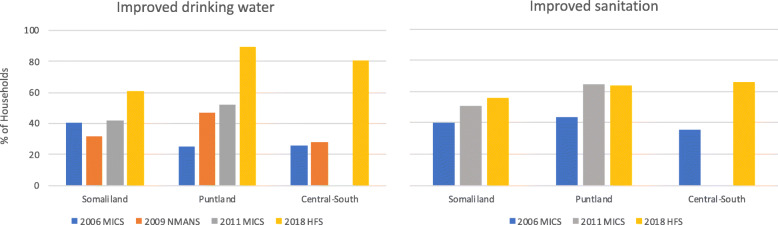
Coverage of improved water and sanitation over time, by zone

Coverage of improved water and sanitation appears to have generally increased over time in all zones, and by 2018, the Somalia High Frequency Survey indicated that Central-South had the highest coverage of improved sanitation, and the second highest coverage of improved drinking water, after Puntland (Fig. 4).

While the available quantitative data provide some indication of geographical differences in intervention coverage at particular time points, little can be inferred from these data about change over time, and even less about the drivers of such change. However, the results of our qualitative analyses provide further insight into the ways in ongoing conflict in Somalia as well as the humanitarian health response in Somalia have affected coverage of RMNCAH and nutrition interventions during the study period.

### Qualitative findings

We first summarise the array of RMNCAH&N interventions that respondents highlighted when reflecting on their organizations’ operations in Somalia during the study period. We then outline the key underlying sociocultural and other contextual factors that respondents identified as those most strongly affecting the planning, execution and effectiveness of the humanitarian health response in Somalia. Next we present the most salient operational influences on humanitarian actors’ programme planning, and how they coordinate with others, and then the key intervention delivery barriers, along with reported strategies to address these barriers.

### The prevailing context of RMNCAH&N service delivery in Somalia

#### Delivered interventions

Our data from interviews with a range of actors operating both nationally and in Bay region or Mogadishu indicate that the delivery of RMNCAH&N interventions since 2000 varied greatly around the country. Generally, the organizations that our respondents were affiliated with provided or otherwise facilitated some combination of antenatal care, postnatal care, and delivery by skilled birth attendants. Expanded Programme on Immunisation (EPI) activities and malnutrition screening for children were also provided by many of these organizations. Provision of HIV (VCT and treatment) and TB services were also reported by multiple respondents, while the provision of health or nutrition services targeted specifically at adolescents was reported by respondents from only two organizations.

#### Sociocultural context

In addition to the various operational issues affecting organizations’ program planning and service delivery discussed below, our respondents also highlighted several underlying sociocultural factors in Somalia that have necessarily influenced the humanitarian health response. Clan dynamics, ethnic discrimination, and particularly inter-clan clashes, was reported as one such factor complicating the delivery of health services, often limiting humanitarian organisations’ access to vulnerable populations.

Women’s disempowerment with respect to health care-seeking and-decision making in Somalia was another important issue raised, affecting women’s access to services. For emergency C-section, for example, respondents indicated that the prevailing norm is for women or providers to seek the consent of a male family member or clan leader, creating delays that can prove fatal to both the mother and the unborn child.

Our respondents also highlighted resistance to hospitalization for some conditions, creating further barriers to appropriate care. In-patient care for severe acute malnutrition was cited as one example,

“ … *because it is not generally seen as a medical issue that requires admission. ‘Give me food and I can … I can deal with it’.”*

*-INGO respondent*

Sociocultural barriers were also highlighted by some respondents as influencing the demand for vaccinations. Several respondents noted how limited health-related knowledge in the population contributes to poor health-seeking behaviour and demand for services.

### Operational influences on humanitarian actors’ RMNCAH&N program planning and intervention prioritization

#### Assessing existing and emerging population needs

Our respondents cited the importance of conducting surveys and assessments such as EPI coverage surveys, SMART (Standardized Monitoring and Assessment of Relief and Transitions) surveys, KAP (knowledge and practices) surveys, needs assessments, and other baseline and endline surveys to identify the needs of their target populations in order to effectively prioritise interventions and plan programs. One Ministry of Health (MOH) official stated that,

“ … *results from the assessments have great impact on our interventions, for example, they affect budget allocation and prioritization of interventions locations. Also the advocacy for funding is determined based on those results received from those assessments*.”

The ongoing 2018 Somali Demographic and Health Survey was anticipated by multiple respondents as a useful data tool to help fill the persistent data gaps in Somalia. It was highlighted that while large-scale data collection in the country is usually undertaken by large NGOs or UN agencies along with the government, and that joint assessments are often conducted by consortia of UN agencies under the auspices of the UN’s Office for the Coordination of Humanitarian Affairs, data collected by NGOs alone are generally available only internally, for their own prioritisation initiatives. These surveys often also have a limited geographic scope, while the DHS will cover national figures.

Difficulties in accurately assessing current needs were compounded by the effects of the unpredictability of the conflict situation in Somalia, including sudden and large influxes of displaced populations as well as infectious disease outbreaks and epidemics. Along with recurrent natural disasters, different population needs sometimes emerged quickly, requiring adjustment to existing programming priorities. Planning for the delivery of services to displaced populations was especially difficult, given the mobility of this group. The droughts of 2011 and 2017 and the floods in the first part of 2018 were cited by a number of respondents as recent examples of natural disasters causing massive population displacements to urban centres that strained many of the already-limited service that were being provided in those areas. In order to address the emerging health needs, respondents cited the prioritization of establishing Cholera Treatment Centres (CTC) to treat higher caseloads of acute watery diarrhea (AWD) and increased measles case management to limit outbreaks in their respective areas of operations.

#### Donor prioritisation

Respondents highlighted that access to emergency funds and the delivery of specialised services such as CTCs ceased when the crises subsided. One health facility staff stated that,

*“ … during disasters, most NGOs and INGOs came to the hospital to support us, they give us funds, supplies and training, but when the disasters were resolved, we do not focus on recovery or development action because the funds end so the most activities are also stopped. So the people complain about low services.”*

While recognising the need to address urgent emerging needs, several respondents highlighted their frustrations with being unable to also address the longer-term concerns of the population. Some reported that prioritisation of key interventions was often mandated by a donors’ priorities, which focused on providing immediate humanitarian assistance rather than development. One international NGO staff member noted that,

*“ … funding has been affecting the way we do business mainly sometimes we have to focus on humanitarian needs, life-saving interventions and not the development interventions which is a big challenge in Somalia. If organisations could focus on development interventions, then we would be able to move somewhere but for twenty years down the line organisations are still … are still trapped on humanitarian delivery of service.”*

Our respondents highlighted that the health system was primarily funded through donors including the US government, the European Commission, and the governments of the UK, Germany, Italy, Sweden, Turkey and Qatar; and that these donors played an important role in determining what health services were provided in the country. This occurred in several ways, such as requiring gender parity in staffing, restricting operations in non-state controlled areas, earmarking funds, and limiting the types of services that were funding. Not all organizations felt these restrictions equally however, and respondents from well-funded international NGOs contended that they were in a financial position to refuse funding if the conditions did not align with their organizations’ priorities.

#### Insufficient funding levels

Nearly all of our respondents, whether UN, INGO, LNGOs or even government representatives, cited financial constraints as a key influence on their program planning. Participants noted that, with the exception of relatively high resource mobilisation to respond to the droughts of 2011 and 2017, funding in Somalia has been insufficient in recent years. Several respondents noted that “donor fatigue” seemed to have set in when looking at funding availability for Somalia; this notion was attributed to the growing number of emergencies around the world, with key informants mentioning South Sudan, Yemen and Syria as examples of ongoing crises that were garnering more attention in recent years.

*“But funding is a big issue. It’s been a long, drawn conflict setting, donors have got fatigue. They may not be willing to continue supporting, so it’s always less than 50% funding available to Somalia health sector in each and every single year.”*

*-UN agency employee*

The winding down of the Joint Health and Nutrition Programme (JHNP) in 2016, a five-year multi-donor programme to improve the health and nutrition indicators in Somalia using a pooled fund, exacerbated the consequences of funding shortages by renewing the fragmentation of the funding landscape after the end of the programme:

*“When that funding had stopped there was a hiccup. So each and every UN agency went to donors separately and tried to secure some funds.”*

*-INGO respondent*

Moreover, in the context of insufficient funding, ongoing insecurity meant that humanitarian actors still had to contend with the higher costs of delivering services to conflict-affected populations in Somalia. Practices such as remote management, wherein organizational operations are coordinated from neighbouring Nairobi or from other large cities in Somalia, had been employed for many years in Somalia due to the insecurity and limited access to certain geographies. These practices, however, increased operational costs for organizations. In addition, security costs, transportation of goods via air to avoid roadblocks and looting also drove up the costs of service delivery.

As a consequence of funding gaps, several respondents reported having to deprioritise interventions they had previously been providing, including secondary health services, free caesarean section for poor beneficiaries, cholera treatment, supplementary feeding programmes, food vouchers and MAM management. Other strategies for dealing with funding shortages included the wider use of cost-effectiveness as a metric for prioritising, with some respondents reporting an increased focus on presumably more cost-effective preventive interventions, including health promotion activities such as messaging to improve health-seeking behaviours and promote access to available services. Among these, the promotion of appropriate infant and young child feeding (IYCF) and exclusive breastfeeding was prioritised by many actors, considered to be high-impact and low-cost. The packaging or bundling of multi-sector interventions was another proposed response to funding shortages, with additional benefits for the communities reached:

*“Putting together all programs into one can help in cost-effectiveness. For example, if every program stands on its own, it would need a lot of financing. However, if we integrate health, nutrition and WASH, you can significantly reduce cost. It is better for the programs logistically and financially. So combining those intervention can have a huge cost benefit. The cost will be decreased, the staff will become effective and it is also advantageous to the community because every team is made of health, nutrition and WASH members.”*

*-Local NGO employee*

Reproductive, maternal, newborn and child health services were most often packaged with nutrition services. However, some NGOs reported packaging health, WASH, and education services as part of a multi-sectoral response. However, many of these respondents were from large INGOs with long-standing presence in the country who likely benefitted from multiple funding streams and had institutional capacity to internally integrate programming across sectors. Finally, securing resources from the Somali diaspora was highlighted by a few respondents as another means of bridging funding gaps to maintain programme priorities.

### Coordination of humanitarian health actors

According to the respondents, the humanitarian response in Somalia was mainly coordinated through UN and international agencies leading relevant humanitarian clusters. Humanitarian response in Somalia falls under the Humanitarian Country Team (HCT) framework, with UN or international agencies leading relevant humanitarian clusters. In Somalia, the health and nutrition clusters are led by the World Health Organisation (WHO) and UNICEF, respectively, in collaboration with the federal and state-level ministries of health. Respondents described the primary purpose of the health and nutrition clusters as information sharing fora to update participants on the month’s or quarter’s activities, and also as coordination bodies, particularly during emergency periods, to avoid duplication of efforts. The cluster structures were not necessarily perceived by respondents as fora where decision are made by participating organisations, but rather where cluster coordinators and deputy coordinators, in conjunction with higher-level convening bodies, mandate the direction of the efforts of the respective clusters.

Our respondents highlighted that, over time, the coordination of the health sector response has changed, with increased engagement of local organisations and with the development and strengthening of ministries at both the federal and state levels. Collaboration between the government and the UN agencies has increased, with the government taking on an increasingly greater role in the priority-setting and monitoring of humanitarian health activities in the country.

*“Yes, the host government takes the decision where we support, the places we support and also the NGOs we support. So it is for the host government to decide where and who we are going to support.”*

*-UN agency employee*

The interviews highlighted the government’s role both at national and at state level in collecting and storing data; setting priorities for humanitarian partners in Somalia, including the prioritisation of underserved areas in the country to counter the clustering in hubs where many actors operate; and facilitating the clearance and quality assurance of commodities and supplies being brought into the country.

Our informants also highlighted the need for a more formalised approach to coordinating the role of the private sector in humanitarian response. Engagement of the private sector by the government through formal public-private partnerships still remains minimal, despite the sector’s large role in providing health services in Somalia.

*“ … as a government, we didn’t have a contracting capacity with the service providers – public and private providers both. Second thing is we still giving priority of establishing public services. So … but currently we have conducted some mapping exercise of the private facility and recognise how large private service providers are and what they are contributing.”*

*-Ministry of Health employee*

### Mitigating barriers to RMNCAH&N service delivery

According to our respondents, the complex operational context in Somalia has produced a number of bottlenecks that affect the delivery of services throughout the country, including chronic supply and human resource shortages, poor infrastructure, and limited access to highly vulnerable populations, all against the backdrop of intense insecurity.

#### Health and nutrition supplies

Respondents highlighted that supplies shortages were very common in Somalia, with challenges related to both procurement and transport. Local procurement in Somalia is limited due to organisations’ internal procurement protocols to ensure quality, with supplies generally sourced regionally, in procurement centres in Kenya, or internationally, from European procurement centres.

Several of our respondents reported that, in their experience, supplies for severe and moderate acute malnutrition were provided exclusively by UNICEF and WFP, respectively, with reproductive health supplies being supplied by UNFPA across the country. Transporting supplies was also difficult, as a result of the scarcity of convoys and the increased risk of ambush and looting of supplies. Many respondents particularly noted essential medicines, vaccines and nutritional supplies as the ones suffering shortages but some participants indicated that because the nutrition domain was better funded by donors, stock-outs of nutritional supplies were less frequent.

To address procurement and supply challenges, respondents highlighted the use of internal borrowing mechanisms from other operational areas within their own organisations, or requesting additional supplies from regional office or headquarters. Often, the airlifting of supplies was employed despite the high costs associated with this.

*“Almost all supplies are delivered through air base, there is no road transport at the moment in Somalia, mainly in the South. These are key issue to consider when prioritising is done.”*

*-UN agency employee*

Forecasting and the utilisation of buffer stocks were other actions undertaken by organisations to mitigate the effects of chronic supplies shortages during the implementation cycle.

*“We plan to utilize the resource we receive for as long a time as possible. We have a stock report that tells us what we have and what we need. We always compare these two, and before we have a stock out, we call for immediate supply replenishment.”*

-Local NGO employee

Beyond the internal mitigating mechanisms used, respondents also reported informal borrowing between organisations in the same district or region, in addition to the referral of beneficiaries to nearby facilities where the required supplies may be available.

Respondents noted that the government had limited capacity for procurement and that almost the entirety of the government supply came from international donors. A federal MOH staff confirmed this, and while acknowledging the challenges around “inadequacies and unequable distribution”, added that the Supply Chain Management Unit has now been created within the ministry to “take some role on forecasting of supplies needed and they are going to take some role on distribution and monitoring of the supply availability at the facility.”

#### Human resources

Multiple participants cited the lack of skilled, qualified health workforce as a main issue that affected the delivery of health and nutrition services. Although human resource shortages were a concern for most health programs, this was particularly highlighted for positions that required surgical expertise. In one instance, a larger international NGO had been unable to provide CEmONC services for a period of 5 months because they were unable to identify a gynaecological surgeon to provide the comprehensive package. Another respondent from a large NGO in Somalia noted the need for fistula care, particularly for adolescent girls with early first pregnancies, adding:

*“In Somalia it’s very hard to get the surgeon who can repair the fistula. So in most cases we have to work with other organisations and maybe bring a surgeon from outside. Maybe from Kenya, maybe from Ethiopia, maybe from the US to come and repair the fistula. So I think that is one area where the entire country is struggling with human resource, yeah. Repair of obstetric fistula is a big problem.”*

*-INGO respondent*

Retention of staff in rural or peri-urban areas poses a problem, as most skilled health workers prefer to work in urban centres. To deal with this shortage, many organisations have developed hiring practices and retention mechanisms to ensure staffing is sufficient to implement activities. The primary strategy identified by participants was to recruit staff connected to the area in which the services are to be provided. One INGO staff explained:

*“We sit with the community, we talk to them and then they were able to get their people in Mogadishu who are well educated, who are qualified, who can easily apply for a job and are able to come to that village, and work without any fear of security or anything. So through networking and coordination with the community we were able to manage the human resources issue.”*

*-INGO respondent*

The importance of providing competitive salaries and training opportunities was also highlighted as a strategy for attracting and retaining qualified candidates:

*“It’s very difficult but we tried to encourage our implementing partners to retain through building the capacities, through training, to have … human resources policies that every year they increase the salary like 10% or 5%.*

*-UN respondent*

The unintended consequences of this strategy for the government were also mentioned, however, with the government finding it difficult to compete with INGOs in attracting qualified candidates. Differences in salary levels between INGOs was also cited as a concern, with implications for recruitment and also for increasing tensions between NGOs.

Task shifting has also been employed by NGOs both local and international, UN agencies and the government in Somalia to deal with the shortages of health workers in the country. In particular, the use of midwives and community health workers was cited as a strategy to deal with shortages of doctors. Respondents also reported on efforts underway to reduce human resource shortages, with one example being UNFPA’s midwifery schools now open throughout the country.

*“Now we have many institutions and universities and many people educated so the health staff are many. So far the opportunity [for] getting health workers, especially midwifes, were too small. It occurs to [advertise] one position three to four times, that you cannot found midwifes, but now condition is good; it’s available because many schools are producing midwifes including UNFPA school of midwifery.”*

*-Local NGO employee*

*“So the plan is to establish a system that registers the health workers, the professionals and the community and then give licensing and accreditation to both the universities, health facilities and professionals. So that it’s something as a priority but currently we don’t have a system in place that registers the health workers.”*

*-Ministry of Health Employee*

Finally, multiple respondents commented on the roles of clan affiliation, community pressures, and political connection of potential recruits as sometimes affecting the hiring of staff for their organisations.

#### Infrastructure

Limited and insufficient infrastructure was cited as a key barrier to service delivery by many respondents. One participant from a large INGO explained how their organisation couldn’t provide hospital-based services until a new hospital wing could be constructed or repurposed from existing infrastructure.

Other respondents commented on the existing infrastructure being disproportionately clustered in and around urban centres, leaving rural populations without access to health services. The need for alternative delivery platforms in such settings was discussed. Several respondents highlighted the importance of mobile clinics and teams which visit remote areas in need of services. However, the inadequacy of such platforms for delivering sufficient care was also acknowledged:

*“The other thing is the availability of infrastructure, health facility infrastructure in the rural districts. So … there is limited service delivery [in] the rural areas. The only option we try is to maybe explore on mobile … mobile and outreach services but it’s not adequately funded and it’s not sustainable. Sometimes we provide mobile and outreach services to the rural areas but the population is underserviced.”*

*-Ministry of Health official*

#### Security

Ongoing insecurity was evident as an overarching theme throughout our interviews, affecting humanitarian actors across the country, particularly in terms of accessing areas outside of government control, the transport of goods and supplies by land, and the mobility and safety of health workers. The vast majority of respondents identified the perpetrators of the violence as “extremists” or “non-state actors”, with the fear of intrusion by these groups highlighted by many respondents.

Respondents highlighted large disparities in safety in certain geographies. Many participants highlighted insecurity as resulting in the temporary closure of facilities and the postponement of mobile and outreach services in what were dubbed by several respondents “last-mile” villages, those where infrastructure was non-existent or non-functional and outside secure geographies.

*“But sometimes what can happen is that Al-Shabaab may totally interrupt the whole services. They attack – like outreach services – they attack and abduct with the staff; they totally shutoff what is going on there.”*

*-Ministry of Health employee*

Violence against health workers and staff was mentioned often by respondents, affecting organisations’ ability to staff projects, particularly in remote areas and areas where active conflict persists. One local NGO staff in Bay region noted that “there were recurrent incidents of kidnapping and bothering of humanitarian workers”, echoed by another LNGO staff in the region who stated that they “[remember] up to three instances [where] someone was targeted during this year. Some of them aid workers – mostly health workers – have been detained and finally a ransom had been paid”.

To address the very fluid security context organisations operate in, respondents highlighted the importance of formal and informal protocols to gather information and assess possible threats to staff and facilities. Formal methods include the utilisation of dedicated security staff or programming staff to monitor the security situation, staff meetings before the start of the work day, and ensuring that they limit the presence of international staff in-country to minimize their exposure to risk and account for a rapidly changing situation. One respondent from a large INGO described the importance of their organisation’s messaging system to keep updated about the security situation:

*“ … [messages describing] … information about accessibility of city, whether there are road blocks or attacks had happened. It covers situations of the previous day and the current day. It tells the staff whether it is suitable for work or not, so the staff get that message before they come out from their homes.”*

- *INGO staff*

Another frequently used method of gathering information was from the community – whether from staff hired in the community, community liaisons or the beneficiaries themselves.

‘At field level, the responsibility of liaising with local leaders for access to communities in operational areas was often left to local implementing partners or national staff. Respondents described how their organizations coordinated with clan leaders, IDP camp leaders and community elders as focal people to negotiate with to provide services, and many respondents also insinuated that there was a need to coordinate with armed non-state actors. Respondents indicated that covert fees are a reality in-country to secure safe passage for supplies transported by road, though no respondent stated outright that the payment of covert fees was practised by their organisation. Carried out by international staff, such covert payments and perhaps even negotiation itself may put organisations,

“in breach of some of the international regulations like counterterrorism regulation, et cetera. So I think the problem is – you know – some of these funding are constrained by international regulation which make it … for example, illegal to work in certain areas or to provide assistance in certain areas because for example some of the assets might go in the hands of prescribed organisations, willingly or unwillingly And I think agencies don’t want to take that risk, and they do work in those areas.”

-Local NGO employee

## Discussion

Our study findings highlight the complex and often challenging informational and operational contexts that have informed the ongoing humanitarian health response for women and children in Somalia. At least a few key issues merit further discussion here, including data and funding availability, the various forms of risk transference historically practiced by international actors in this insecure environment, the evolving role of the Somali diaspora in the humanitarian health response, and the expanding capacity of the federal government to shape that response.

In the context of a protracted conflict, the local health authorities in Somalia face the double burden of rebuilding the health system while also dealing with acute health emergencies caused by ongoing insecurity as well as natural disasters. Humanitarian actors rely heavily on rapid needs assessments for program planning and intervention prioritization in this context, but there is still a clear lack of high-quality, representative quantitative data available to inform critical decision-making in Somalia. The country’s last census was conducted in the 1970s under Siad Barre’s regime [12], with demographic and other relevant information coming, since then, mostly from household sample surveys such as the 2014 Population Estimate Survey of Somalia led by UNFPA or the 2006 and 2011 MICS surveys supported by UNICEF, none of which were able to access all areas of the country. Regular post-rains reports from the Food Security and Nutritional Analyses Unit (FSNAU) in Somalia, supported by UN’s Food and Agriculture Organization (FAO) are the only recurrent data available spanning the entire territory. Several senior UN staff, government officials and others that we interviewed highlighted the importance of up-to-date data to ensure prioritisation and programming is undertaken effectively, and that they were eagerly awaiting results from the ongoing Somali Health and Demographic Survey (SHDS). The SHDS is being carried out in conjunction with MOH at Federal level, supported by UNFPA, and aims to ensure that capacity building of focal staff occurs for future data collection exercises.

In addition to insufficient funding, respondents highlighted the nature of funding and its focus on humanitarian and emergency response versus development programming as another major issue, with the inability of organizations to secure longer-term funding for sustainable recovery in Somalia impeding further development of the health system. Much of the response has necessarily focused on short-term, high-volume activities to address critical emergencies such as recurrent floods and droughts that result in food insecurity, acute malnutrition and disease outbreaks, as well as continuous displacement to urban centres such as Mogadishu, Baidoa and Kismayo. Only some international NGOs – and even fewer local organisations – have sustainable and multiple funding streams and the institutional capacity to also provide longer-term, multi-sectoral programming that could improve community resilience to such events.

The need to link emergency funding and programming to longer term development is the dominant position in the contemporary discourse on humanitarian action, linked to the ‘localisation agenda’ promoting the building of local capacity to ensure that humanitarian responses are locally led [13]. The transference of risk from international to local actors is a sometimes invoked as corollary however, [14] particularly in the context of conflict and insecurity, and our study participants highlighted various existing examples risk transference in the Somali context.

In part because of funder regulations as well as internal regulations with which international humanitarian agencies need to comply, local implementing partners are often the ones who must shoulder the responsibility of liaising with non-state actors to negotiate, and sometimes pay for, access to areas under their control and to ensure that service provision continues in those areas. Roadblocks and blockades are a reality in parts of Central and South Somalia, greatly affecting the main routes used in the country to transport goods and access rural districts in the country and serving as a critical funding stream for non-state actors. A 2011 report by the UN Monitoring Group estimated that these and other payments by humanitarian agencies to Al-Shabaab translated into yearly income between $70 and $100 million dollars [15].

Risk transference was also recognized by some in organizations’ strategies to hire local staff for frontline service delivery in Somalia. The leveraging of community networks was noted as being important in ensuring the safety of staff working in particular areas, acting as a supplementary alert system to any incidents or risks arising in the area, and both local and international NGOs noted that hiring health workers from the communities they serve can improve retention and helps ensure continuity of service provision through funding gaps and periods of insecurity. Community leaders also act as informal recruitment mechanisms, sourcing qualified staff from their networks to enable humanitarian organisation to staff hard-to-reach and rural areas. Nonetheless, while international humanitarian staff have typically maintained only an intermittent presence within Somalia, local staff based in situ have much higher exposure to violence, abductions and other insecurity risks. In 2018 alone, over 110 violent incidents were recorded which directly impacted humanitarian operations across the country, though the true number is likely to be higher due to non-reporting [1]. Moreover, the humanitarian health response occurs within a particular sociocultural context, wherein clan dynamics and inter-clan clashes can also inform service delivery. Navigating clan dynamics can be particularly complex for humanitarian organizations who must balance working closely with clans in the geographies they serve, while taking care not to alienating other clans.

Despite the increased insecurity risks borne by local actors, the broader diaspora community was identified by respondents as playing an important role in health service delivery in Somalia, with Somalis from neighbouring countries and around the world returning to Somalia over recent years to contribute both as local health care providers at facility level and as programming staff. The diaspora’s importance as an additional source of financing to tackle funding gaps and infrastructure construction and rehabilitation was also noted. Since the start of the conflict in 1991, diaspora remittances to Somalia have been estimated at around $11.2 billion [16].

Remote management from hubs like Nairobi and from large cities such as Mogadishu has been useful in allowing international actors’ in-country presence while also ensuring the safety of their staff, despite the associated high costs. Currently, both the UN and large INGOs have begun or have completed the process of transferring their bases of operation from Nairobi to Mogadishu, a move that was heavily championed the Ministry of Planning, Investment and Economic Development. The Minister suggested that those organizations not complying with the new directive would not be allowed to operate in the country and their registration would be withdrawn [17]. In addition to addressing the disproportionate risk burden experienced by local actors, greater in-country presence of international humanitarian actors may reduce operational costs and improve the efficiency of service delivery in the country.

While the provision of many basic services in Somalia, including health and nutrition services, has historically been led by local and international NGOS throughout Somalia’s ongoing conflict, the coordination of the health and nutrition clusters has now evolved from an exclusively UN-led mechanism to one that includes a more active role for the Federal Ministry of Health (MOH) in monitoring and coordinating activities. Humanitarian agencies have increasingly recognised the evolving MOH role and work towards the MOH’s goals of strengthening governance and institutional capacity, creating sustainable health financing mechanisms, [18] increasing per capita health expenditure and reducing maternal and child mortality [19]. Nonetheless, because of its limited capacity, the MOH continues to rely on MOUs with individual organisations and, presently, no centralised system of MOH oversight exists to monitor activities by partners. The implementation of a National Disaster Management Policy spearheaded by the Federal Ministry of Humanitarian Affairs and Disaster Management is one of the new efforts emerging in recent years to create greater oversight by the Somali government in the coordination of humanitarian response, including the humanitarian health response [20].

### Study limitations

One limitation of this study was the lack of quantitative data available to examine change over time in RMNCAH and nutrition intervention coverage, which may have allowed better synthesis of our quantitative and qualitative findings and a deeper understanding of service delivery patterns and their drivers in the context of the humanitarian health response in Somalia. With respect to limitations related to our qualitative methods, the timelines and logistic constraints of our study required the use of multiple data collectors. Debriefs between different data collectors helped to minimize the impact of multiple data collectors on data quality. The identity of the data collectors and how the study team was perceived may have also played a role in the disclosure of respondents. Moreover, our data collectors were not directly involved in the data analysis. However, close contact between ZA and the study team aided with the analysis of the interviews. Lastly, the generalizability of our qualitative findings is limited.

## Conclusions

Our study characterized the complex context within which humanitarian health actors have delivered RMNCAH and nutrition interventions in Somalia, highlighting actors’ key program planning considerations and strategies for addressing barriers to service delivery, including ongoing insecurity. Several recommendations for future research and action emerge from this work, including the further exploration of government partnerships with private sector service providers to make essential RMNCAH and nutrition services available throughout Somalia free of charge. We also advocate for further research on innovative uses of technology to help ensure that Universal Health Coverage reaches remote and inaccessible areas of Somalia. To further mitigate the shortage of skilled health workers in Somalia, more operational research is needed to study the expanded use of community health workers in this conflict-affected context, especially for providing ANC and PNC services. It is important to also recognize and address persistent gaps in service provision across the continuum, including gaps for certain age groups such as adolescents, as well as the need for longer term development projects, including the building of infrastructure, to enable the provision of health and nutrition services for beyond those including in recurrent emergency response. The humanitarian community must make a concerted effort to ensure that their services comprehensively address the health needs of women and children in Somalia.

## Data Availability

The quantitative data used in this study are available in the public domain. The qualitative data used in this study are not available to the public because the terms of the informed consent to which participants agreed stated that only members of the study team would have access to the data.
